# Examining cognitive load in human-machine collaborative translation: insights from eye-tracking experiments of Chinese-English translation

**DOI:** 10.3389/fpsyg.2025.1570929

**Published:** 2025-11-12

**Authors:** Lei Chen

**Affiliations:** Foreign Languages School, Sichuan University Jinjiang College, Meishan, China

**Keywords:** human-computer translation, eye tracking experiments, rank sum test, data fitting, cognitive load

## Abstract

**Introduction:**

With the development of artificial intelligence and computer science, human-computer collaborative translation (HMCT) mode has gradually become a research hotspot in the field of English translation. The purpose of this study was to explore the cognitive load characteristics of translators in the process of human-computer collaborative translation through eye tracking experiments of Chinese-English translation. Based on a 2 × 2 hybrid design, the participants’ eye movements were analyzed under the conditions of simple, medium and complex texts through two tasks, human translation and human-computer collaborative translation.

**Methods:**

The study involved 30 master’s students or translators in translation who used Tobii Pro Glasses2 to record eye tracking data in real time, focusing on fixation time, regressionness, saccade and fixation point to reveal the impact of different Chinese-English translation tasks and text types on cognitive load.

**Results:**

The experimental results show that the fixation time, the numbers of regressions, fixations and saccades of human translation are significantly higher than those of human-computer collaborative translation, especially in complex text tasks. At the same time, the numbers of regressions and fixation time increased significantly with the increase of task complexity in both groups, and the human translation group showed a higher cognitive load in complex tasks.

**Discussion:**

This study finds that the cognitive load of translators in the process of human-machine collaborative translation shows phased changes, especially when the output quality of machine translation is poor, translators need more cognitive resources to correct. The impact of complex tasks on cognitive load is even more significant, and human translation requires more cognitive effort on the part of translators. Eye tracking data analysis provides empirical support for understanding the cognitive mechanisms in the translation process. For the first time, this study systematically explored the cognitive load characteristics of human-computer collaborative translation through eye tracking technology, filling the research gap in this field in the existing literature. The results of this study not only provide a theoretical basis for optimizing translation tools and designing more efficient translation processes, but also provide a new perspective for cognitive load management in translation teaching and practice.

## 1 Introduction

With the rapid development of computer science and artificial intelligence technology, machine translation (MT) has made significant progress. In recent years, neural machine translation (NMT) technology has made significant breakthroughs in translation accuracy and fluency, and has become a hot topic in the field of translation research. However, although machine translation performs well in dealing with standardized texts and translations in common domains, it still has great limitations when dealing with complex sentence patterns and context-dependent texts (Wang and Chen, 2024). Therefore, many translation researchers have proposed the Human-Computer Collaborative Translation (HMCT) model, which aims to combine the language understanding ability of human translators with the computational efficiency of machine translation to improve the quality and efficiency of translation. The core concept of human-machine collaborative translation is that machine translation is used as an auxiliary tool to provide human translators with initial translation suggestions, and the translator can make corrections, polishes, and adjustments based on them. This model can not only improve the speed of translation, but also alleviate the repetitive work in human translation to a certain extent, and promote translators to deal with complex corpora more efficiently (Pang et al., 2024). However, this process places higher demands on the cognitive load of translators. Translators need to constantly switch their attention, evaluate the quality of machine translation output, and adjust and optimize the language during the translation process, which undoubtedly increases the pressure on the allocation of cognitive resources (Mahsuli et al., 2023).

Cognitive load theory (CLT) indicates that when an individual performs a task, if the task demands exceed their cognitive resource capacity, it will lead to cognitive overload and impair task performance (Cui et al., 2024). In translation processes, especially in human-machine collaborative translation scenarios, translators not only need to complete traditional text understanding and generation tasks but also must evaluate and correct the machine translation output, which significantly increases cognitive load. Therefore, accurately measuring the level of cognitive load in collaborative translation environments and analyzing its influencing factors has become a key issue in current translation research. Eye-tracking technology, as a reliable method for measuring cognitive load, has been successfully applied in cognitive research areas such as language comprehension, reading processes, and translation activities (Kasper et al., 2023). This technology records key indicators like fixation point distribution, saccade frequency, and fixation duration, providing real-time feedback on the cognitive resource allocation of translators during the translation process. Systematic research on human-machine collaborative translation cognitive load using eye-tracking technology not only helps deepen our understanding of how translators process machine translation outputs but also provides important theoretical foundations and practical guidance for optimizing translation tool interface design and enhancing human-machine collaboration efficiency.

Cognitive load theory (CLT) states that when an individual is performing a task, if the task exceeds the capacity of its cognitive resources, it will lead to cognitive overload and affect task performance. In the translation process, especially in human-computer collaborative translation, translators are faced with not only the problem of understanding and generating texts, but also the judgment and correction of machine translation results, which may lead to an increase in cognitive load (Cui et al., 2024). How to effectively measure the cognitive load of translators in collaborative translation and analyze its influencing factors has become an important topic in translation research. As a powerful cognitive load measurement tool, eye tracking technology has been widely used in cognitive research fields such as language comprehension, reading, and translation. Eye tracking experiments can provide researchers with real-time data on cognitive resource allocation during the translation process by monitoring eye movement indicators (such as fixations, number of regressions, fixation time, etc.) (Kasper et al., 2023). Therefore, the systematic study of the cognitive load in the process of human-machine collaborative translation combined with eye tracking technology can not only help to reveal the cognitive mechanism of translators when processing machine translation output, but also provide an important reference for optimizing the design of translation tools and improving translation efficiency.

## 2 Literature review

Human-computer collaborative translation (HMCT), as a translation mode that combines the advantages of artificial intelligence and human cognition, has attracted extensive attention in recent years. In this mode, the machine translation system generates a preliminary translation, and the translator revises and polishes it later, so as to improve the translation efficiency and maintain a high translation quality. Early research focused on the performance evaluation and improvement of machine translation, but with the rise of neural machine translation (NMT) technology, researchers have begun to explore how to combine AI with translators’ linguistic abilities to optimize the translation process and improve overall effectiveness (Baumgarten, 2024); Some studies have pointed out that machine translation still cannot completely replace the judgment and creativity of human translators in the translation of complex texts or professional fields, which also provides a new research direction for human-computer collaborative translation (Chen and Kruger, 2024). Cognitive load theory (CLT) was proposed by Sweller (1988) to explain how an individual’s limited working memory resources can be effectively used when performing cognitive tasks. The theory classifies cognitive load into three types: intrinsic load, extrinsic load, and necessary load. Intrinsic load refers to the difficulty of the task itself, extrinsic load is a burden caused by unnecessary factors, and necessary load refers to the cognitive resources required to complete the task. The cognitive load in the translation process mainly comes from multiple aspects of language comprehension, information processing, and generation (Chang and Chen, 2023).

In traditional translation studies, the study of cognitive load focuses on human translation, exploring how translators modulate cognitive resources between understanding the source language and generating the target language (Wang and Daghigh, 2023). With the development of machine translation technology, more and more studies have begun to pay attention to the cognitive load in human-machine collaborative translation, especially the impact of the quality of machine translation on the cognitive load of translators. As an important tool for studying cognitive load, eye tracking technology can accurately capture various indicators of the translator’s eye movements during the translation process, such as fixation points, fixation duration, and number of regressions, so as to reveal the allocation of cognitive resources. In recent years, eye tracking has been increasingly used in translation research as an effective method to analyze the cognitive load of translation (Ge et al., 2023). Eye tracking data can reflect the translator’s cognitive response to different types of text, such as pause, look back, or even reorganize the language when confronted with a difficult sentence (Odusami et al., 2024). Eye tracking research provides valuable empirical evidence for understanding the cognitive mechanisms of translators in the translation process. In the study of machine-translation-assisted translation, eye-tracking experiments have been used to analyze the difference in cognitive load between machine translation and human translation. For example, Musk and Meij (2024) analyzed the cognitive load of translators when processing machine translation output and human translation through eye tracking, and found that the quality of machine translation output significantly affected the number of reviews and fixation time of translators (Musk and Meij, 2024). Inaccuracies in machine translation output can lead to translators needing to allocate additional cognitive resources when correcting translations, thus increasing cognitive load. Similar studies have shown that factors such as the quality of machine translation results, translators’ language proficiency, and the complexity of the text all affect the distribution of cognitive load (Liu et al., 2024).

Real-time eye movement data collection in collaborative tasks has been achieved through multi-device synchronization technology, providing a new method for evaluating the attention allocation during the human-machine interaction process. Experiments show that the system can effectively capture the pattern of translators’ visual focus shifts in the scenario of collaborative translation, offering data support for optimizing the interface design (Mahanama et al., 2023). A neural machine translation (NMT) optimization framework named LenM for low-resource languages has been proposed. By modeling the length characteristics of the target language, the translation quality is improved. Research indicates that in language pairs with scarce resources, explicitly introducing length constraints can reduce overly short or redundant translations and significantly improve the BLEU score (Mahsuli et al., 2023). The candidate translation selection strategies of online translation tools in EFL collaborative writing tasks have been explored. Based on conversation analysis, researchers have revealed the process by which users screen the outputs of machine translation through critical interaction strategies, emphasizing that human-machine collaboration needs to balance the efficiency of automation and language learning goals (Musk and Meij, 2024). The application of multimodal neuroimaging data in the staging and classification of Alzheimer’s disease (AD) has been systematically elaborated. Through meta-analysis, the performance of machine learning models has been compared, and it has been pointed out that integrating structural MRI and functional imaging features can improve the accuracy of early AD identification (Odusami et al., 2024). The utilization of monolingual data in low-resource NMT has been re-evaluated, and a strategy of dynamic curriculum learning has been proposed to optimize the pre-training process. Experiments have demonstrated that the progressive introduction of monolingual corpora can alleviate the overfitting problem caused by data sparsity, especially with remarkable effects in language pairs with significant word order differences (Pang et al., 2024).

Although previous studies have explored the cognitive load in machine translation and human-machine collaborative translation, most of them focus on the comparison between machine translation and human translation, lacking a detailed analysis of the cognitive load of translators during the human-machine collaborative translation process. In particular, the application of eye-tracking technology in this field is still relatively limited. Existing literature pays more attention to the quality of machine translation output, but lacks a comprehensive analysis of the cognitive load for different types of translation tasks and different translation texts. In addition, existing research has rarely explored how the complexity of translation tasks interacts with changes in cognitive load. This study adopts the framework of the Cognitive load theory (CLT), clearly defining cognitive load as the total consumption of working memory resources of individuals when performing translation tasks, and distinguishing it into intrinsic load (inherent task difficulty), extraneous load (burden caused by the interface/tool), and germane load (effective processing resources). Different from cognitive effort or mental effort, the load is objectively quantified through eye-tracking indicators (such as fixation duration, number of regressions, etc.). Through an eye-tracking experiment of Chinese-English translation, this study aims to fill the above-mentioned gaps, conduct an in-depth analysis of the characteristics of cognitive load during the human-machine collaborative translation process, and explore the relationship between the complexity of machine translation tasks and the cognitive load of translators. Through systematic empirical research, this study can not only provide a new cognitive perspective for human-machine collaborative translation, but also provide theoretical support for the design of future translation tools and teaching methods.

## 3 Research methods

This study aims to explore the cognitive load in the human-computer collaborative translation process through eye-tracking experiments of Chinese-English translation. To achieve this goal, the research designed experimental tasks for participants, eye-tracking experiment settings, and data collection and analysis methods. The following section will detail the participants, design and implementation process of the experiment, as well as the specific steps of data collection and analysis methods.

### 3.1 Participants and experimental design

The participants in this study were 30 postgraduate students or professional translators with some translation experience. Participants included 15 graduate students with basic translation training (average experience = 1.5 years) and 15 professional translators (average experience = 6 years). All participants passed a standardized English proficiency test (CET-6550 or TEM-870) and reported their weekly use of the MT tool. Group differences were controlled for in subsequent analyses. Demographic information is shown in Table 1. According to the preliminary research results, the participants were required to complete two types of Chinese-English translation tasks: one is a human translation task, and the other is a human-machine collaborative translation task. In the human-machine collaborative translation task, the machine translation system will generate a preliminary translation, and the participants need to make corrections based on the machine translation results.

**TABLE 1:** Demographic information.

Variable	Category	Postgraduate students (*n* = 15)	Professional translators (*n* = 15)	Total (*N* = 30)
Age	Mean ± SD	24.2 ± 1.8	28.6 ± 3.2	26.4 ± 3.1
	Range	22–27	25–35	22–35
Gender	Female	10	8	18
	Male	5	7	12
Education	MA in translation	15	6	21
	Other humanities	0	5	5
	Technical fields	0	4	4
MT tool usage	Daily	9	12	21
	Weekly	6	3	9

The experiment adopted a 2 × 2 mixed experimental design: among them, the task type (human translation/human-computer collaborative translation) was the inter-subject variable, and the text type (simple text and complex text) was the intra-subject variable. Specifically:

(1) In terms of task types, participants were randomly assigned to two groups: the human translation group only performed human translation tasks, and the human-machine collaborative translation group only performed human-machine collaborative translation tasks;

(2) In terms of text types, all participants are required to handle two types of texts: simple texts (including common grammatical structures and basic vocabulary) and complex texts (including professional terms, long sentences and complex syntactic structures).

During the experiment, participants assigned to the human translation group performed only human translation tasks, while those assigned to the human-machine collaborative translation group performed only human-machine collaborative translation tasks. All participants completed their tasks in 30 min.

This study uses multi-dimensional indicators to determine text complexity, which include: (1) Syntactic complexity (average sentence length, depth of clause embedding, referring to Fredrick and Craven, 2025); (2) Lexical complexity (proportion of technical terms, word frequency level, based on the COCA corpus); (3) Discourse coherence (density of referential cohesion, analyzed using the LSA algorithm). Simple texts are everyday expressions (with an average sentence length of ≤5 words and the proportion of technical terms <5%). Complex texts contain technical terms (≥20%) and long and difficult sentences (with an average sentence length of ≥25 words). This standard has passed the consistency test of three linguistics experts (Kappa = 0.82).

To ensure the validity of the text difficulty classification, the rater consistency test and pre-experimental verification were conducted. First, all texts (simple, medium, and complex) are independently classified by three linguistics experts according to the preset criteria (syntax/vocabulary/discourse complexity). The Cohen’s Kappa coefficient 0.82 indicates a high agreement between experts. Another 10 translators (not involved in the formal experiment) were recruited to score the text difficulty at level 5 (1 = very simple, 5 = very difficult). Analysis of variance (ANOVA) showed significant differences between groups (F (2,27) = 38.6, *p* < 0.001) and *post-hoc* test confirmed that the gradient was expected (simple: *M* = 1.8 ± 0.4; medium: *M* = 3.1 ± 0.5; complex: *M* = 4.3 ± 0.6).

### 3.2 Design and implementation of eye movement experiment

In the experiment, the Google NMT API (2024 version) was used to generate the initial translation. Its training data covers both general and professional fields and supports context-aware translation. The selection criteria include: (1) It has the highest market share, representing the mainstream technology; (2) The API has high output stability, which can avoid the version differences of localized tools. The system parameters were set to the default values to simulate the daily usage scenarios of translators. The translation results were uniformly presented after preprocessing.

**FIGURE 1:**
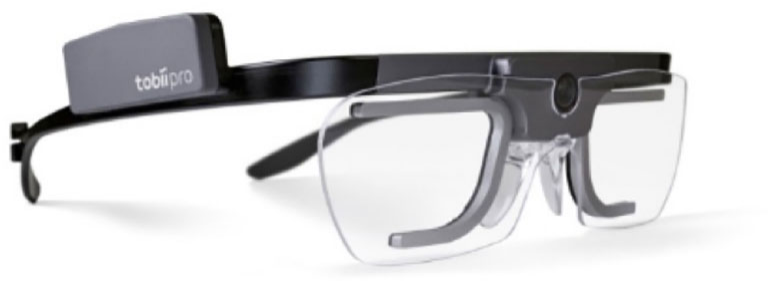
Tobii Pro Glasses2 eye tracking headset.

In this experiment, the Tobii Pro Glasses 2 wearable eye tracker (sampling frequency: 100 Hz) was used to record the participants’ eye movement data in real time. This device captures the eye movements through a head-mounted infrared camera and supports data collection in an environment with natural head movements, which meets the requirements for dynamic observation of the translation process. The design of the eye movement experiment is based on the cognitive load characteristics during the translation process, focusing on the changes in the translator’s eye movement responses during translation, especially the differences under different text types and task conditions. The eye tracker used in the experiment is the Tobii Pro Glasses2 eyewear eye tracker. The Tobii website provides a user manual for this model eye tracker, which includes specifications and sensor information for the eye tracker, as shown in Figure 1.

The environment was a quiet, distraction-free room, with participants seated 60 cm away from a computer monitor, and the eye tracker mounted under the screen. Participants received simple training before the experiment to familiarize themselves with the experimental environment and operation procedures of eye tracking. Before each experiment, participants will need to calibrate to ensure that the eye tracker is accurately recording eye tracking data. Before the start of each experimental task, participants are given clear instructions for the task and the translated text is presented on the screen. In a collaborative translation task, the results of the machine translation are displayed below the source language text, and participants need to make modifications on this basis. The eye tracker continuously records the participant’s eye tracking data, including key metrics such as fixation points, fixation time, number of regressions, and saccades.

The experimental texts used include news reports (10 pieces, with an average sentence length of 12.3 words ± 2.1 and the proportion of technical terms being 3.8%), moderate texts (10 pieces, with an average sentence length of 18.7 words ± 3.5 and the proportion of technical terms being 12.4%), and texts sourced from medical theses (10 pieces, with an average sentence length of 26.9 words ± 4.8 and the proportion of technical terms being 23.1%). All texts were classified by three linguistics experts according to the criteria in section 3.1 (Kappa = 0.82), and the difficulty gradient was verified through a pre-experiment (*p* < 0.01).

In this experiment, a Tobii Pro Glasses2 wearable eye tracker (sampling frequency: 100 Hz) was used to record participants’ eye movement data in real time. The device captures eye movements through a head-mounted infrared camera and supports data collection in a natural head-moving environment, which meets the requirements of dynamic observation in the translation process.

### 3.3 Data collection and analysis methods

In this study, we employed the Wilcoxon sign-rank test for statistical analysis. The Shapiro-Wilk test (*p* < 0.05) confirmed that the eye-tracking data followed a non-normal distribution. With a sample size of 30 (*n* = 30), some indicators contained extreme values. For inter-group comparisons, we provided both *p*-values and *r*-values (*r* = Z/√N) to measure effect sizes; for intra-group comparisons, we used Wilcoxon effect sizes (*r* = Z/√N). Effect sizes quantify the practical significance of differences (e.g., *r* > 0.3 indicates moderate effects), avoiding reliance solely on statistical significance. Text materials were categorized into low, medium, and high complexity levels (low, medium, high) based on predefined criteria. However, to eliminate order effects and focus on specific research questions, during experimental design and data analysis, we systematically grouped texts with medium complexity into “simple” or “complex” categories based on linguistic features. These features included: (1) Lexical density, defined as the proportion of content words (nouns, verbs, adjectives, adverbs) to total words per 100-word segment; (2) Syntactic complexity, measured by the average number of clauses per sentence; (3) Mean sentence length, calculated as the total number of words divided by the number of sentences; (4) Lexical diversity, represented by the Type-Token Ratio (TTR), which is the ratio of unique words to total words in the text. This resulted in two distinct main categories at the final analytical level: simple (including low and partial medium) texts versus complex (including high and partial medium) texts. Thirty participants were divided into six groups of five, using a Latin square design to balance task sequences. Participants in each group completed manual translation (MT) and human-computer collaborative translation (HMCT) tasks under differentiated sequences. Within each group, the presentation order of texts classified as “simple” and “complex” based on the final definitions was randomized. Prior to the experiment, a 5-min warm-up task using non-experimental text was administered to stabilize participants’ states. The interval between two experimental tasks was set to ≥24 h to prevent fatigue effects. Task sequence allocation was achieved through a random number table, with double-blind control implemented throughout the process.

**FIGURE 2:**
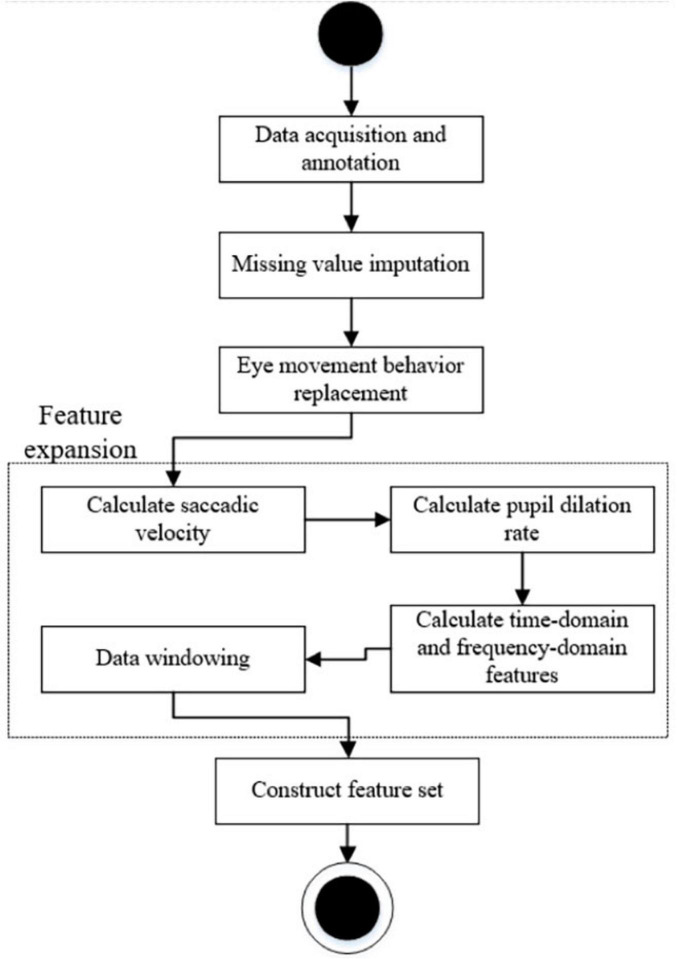
Overall process of eye movement data processing.

The data was collected using Tobii Pro Studio, an eye tracking software that provides participants’ eye trajectories and associated eye tracking metrics. Each experimental task will generate a set of eye tracking data files that record all fixations, regressions, and saccade behaviors of the participants during the translation process. The data file will be exported and further processed. The process is shown in Figure 2.

**FIGURE 3:**
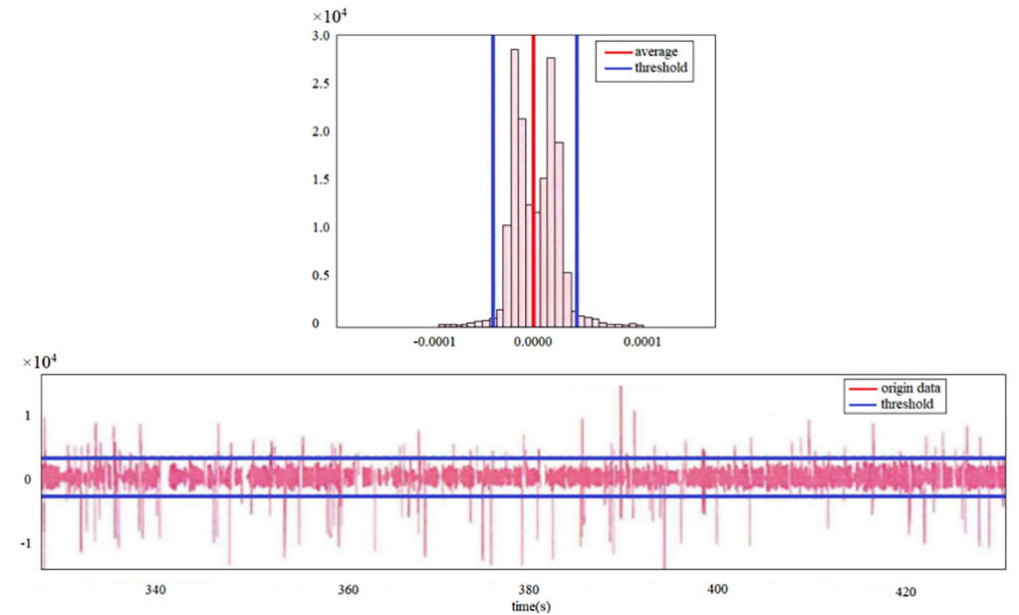
Missing values for movement change data.

There are many missing values in the collected eye tracking data, either due to missing eye tracker sampling or due to blinking. For subjects whose sampling frequency is too low, their data have been discarded; For the inability to find the eye caused by blinking, the missing value needs to be processed appropriately to facilitate the subsequent data processing process. Through the image of the pupil data, it can be found that when the participant blinks, the pupil is occluded, the eye tracker sampling is abnormal, and the pupil diameter data will first decrease rapidly, and then produce a missing value (Figure 3). If the missing values are filled directly by the commonly used linear imputation method, the pupil data value of the imputed part will be low, while the imputation results will be biased if the imputation or mean imputation is used. Since the pupil diameter data changes rapidly when blinking occurs, the rate of change of pupil data is used as an indicator to identify the occurrence of blinking, and the calculation method is to use the first-order forward difference of pupil data as the rate of change of pupil data.

To identify the occurrence of blinking, it is necessary to determine the value of the blink rate change. The distribution of the rate of change in pupil diameter is mostly concentrated within a certain range, with fewer data points beyond this range and significantly away from the mean. To identify the occurrence of blinking, the IQR method (Interquartile Range) is used to identify outliers (Equations 1–3):


$$I\,Q\,R=Q_{3}-Q_{1}$$ (1)

$${\text{Threshold}}_{1}=Q_{3}+\text{k}•I\,Q\,R$$ (2)

$${\text{Threshold}}_{2}=Q_{1}-\text{k}•I\,Q\,R$$ (3)

In the formula: Q3 is the upper quartile, Q is the lower quartile. In the original IQR method, k is set to 1.5 to detect outliers. When *k* = 1.5, it can efficiently detect outliers but cannot accurately detect blinks. Therefore, it is appropriate to reduce k. After trying, it was found that when *k* = 0.7, the detection effect on blinks is better.

After determining the *k*-value, ± 3 × 10−5 can be selected as the value to identify the blink according to the above formula. When a mutation in the rate of change of pupil diameter is identified, 6–8 data before and after the mutation point are replaced with missing data, and then linear interpolation is performed.

It can be seen from Figure 3 that the IOR method has a small impact on the mean of the original data, and it is possible to roughly identify blink data from the original data when the eye tracker cannot detect blinking. Data analysis was performed using SPSS.24. First, the eye movement data was initially cleaned and preprocessed, removing invalid or interfering data (such as blinking, external interference, etc.). Then, the main eye movement indicators under different tasks and text types, such as fixation duration, number of saccades, and number of fixation points, were calculated separately. Finally, the independent sample *t*-test was used to analyze the impact of different task types on eye movement indicators, and the cognitive load differences under different translation tasks were analyzed. In addition to eye movement data, the translation time and translation quality ratings of the participants were also collected. The translation quality was scored by two professional translation reviewers according to a standardized scoring system, with criteria including grammatical correctness, fluency, and completeness.

### 3.4 Indicators and tools for cognitive load measurement

The measurement of cognitive load is the core of this study, and eye movement data is the main indicator of cognitive load. Specifically, the following eye movement indicators are selected as representative indicators of cognitive load.

Fixation Duration: Refers to the time the eyes remain at a fixed position. A longer fixation duration is usually associated with higher cognitive load, especially when encountering more difficult sentences. Regression Count: Refers to the number of times the eyes return from a later position to an earlier one. A higher number of regressions indicates that the translator encountered difficulties in understanding or processing the translation content, requiring additional cognitive resources for information review. Saccade Count: Refers to the number of times the eyes move from one fixation point to another. Frequent saccades may indicate that the translator’s attention shifts rapidly during translation, reflecting dynamic changes in information processing. Fixation Count: Refers to the number of different positions the eyes stop at during the translation process. A higher fixation count indicates the complexity of the translation text or the translator’s in-depth processing of the text.

## 4 Results and analysis

### 4.1 Analysis results of eye movement data

Median was calculated using the Hodges-Lehmann estimator to calculate the median difference between groups and its 95% confidence interval. Eye-tracking data provides us with detailed behavioral patterns of participants during the translation process. By analyzing key indicators such as fixation duration, number of saccades, number of fixations, and the number of fixation points, it can directly reflect the translator’s cognitive process in handling translation tasks.

**FIGURE 4:**
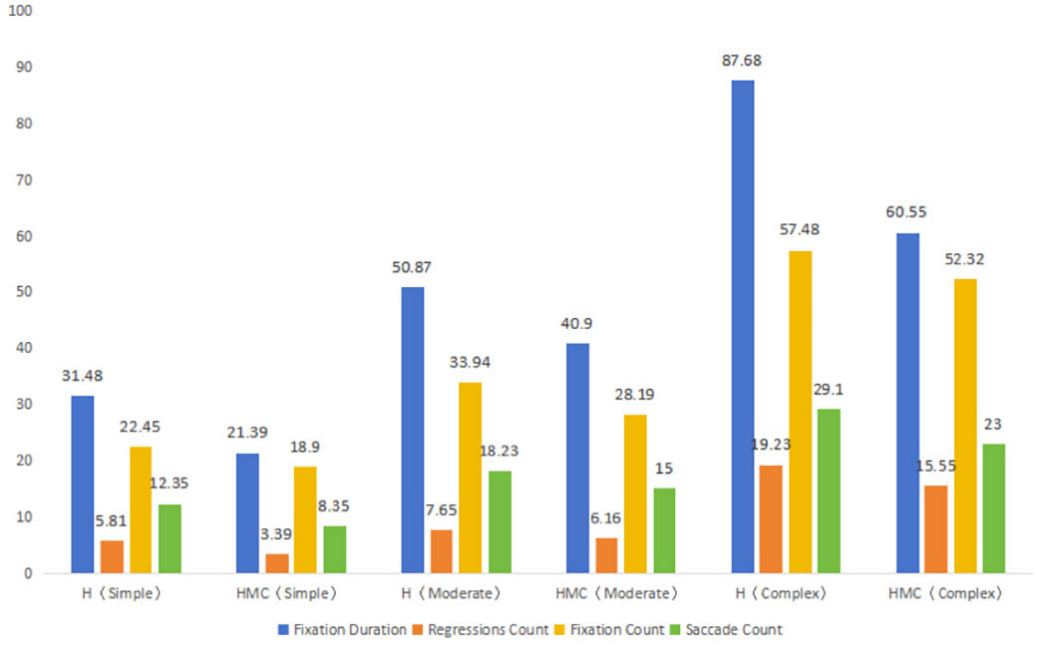
Differences in gaze and saccade-related indicators among two types of tasks.

According to the data in Figure 4, the fixation time of human translation is higher than that of human-computer collaborative translation, regardless of the difficulty of the task, and the fixation time increases significantly with the increase of task complexity. Especially in complex tasks, the fixation time of human translation is much higher than that of human-robot collaborative translation. This suggests that human translation requires more cognitive input, especially in complex tasks, where translators need to pay attention for longer periods of time. Similar to fixation time, the number of regressions was significantly higher than that of human-computer collaborative translation, and the number of regressions increased significantly in both groups with the increase of task complexity. The increase in the number of regressions in human translation, especially in complex tasks, may reflect the need for translators to work on complex tasks with more back-checking to ensure accuracy and fluency of translations. As the difficulty of the task increased, the number of fixations increased significantly in both groups, especially in the human translation group. In complex tasks, the number of fixations in human translation is much higher than in human-robot collaborative translation, suggesting that translators may need to go through the text more extensively and adjust details in complex tasks. The manual translation group consistently exhibited higher eye movement frequency than the human-computer collaboration group. As task complexity increased, both groups showed significant increases in eye movements, with the manual group demonstrating particularly notable growth. This indicates that translators require more frequent shifts of fixation points between text areas when handling complex tasks. Overall, all ocular metrics (fixation duration, eye movement frequency, number of fixation points, total fixation distance) demonstrated an upward trend with increasing task complexity. These findings suggest that complex tasks impose greater cognitive demands on translators, requiring them to invest more time and effort while making more frequent adjustments during processing. Through further analysis of pattern variations across translation phases, we divided the eye movement time series of two groups of translators into three stages based on task duration: early, middle, and late. The early stage showed shorter fixation durations and more frequent saccades, indicating initial rapid text scanning. The middle stage demonstrated significantly extended fixation durations and increased fixation returns, reflecting in-depth processing and comprehension of key information. The late stage featured slightly shortened fixation durations and stabilized saccades, demonstrating pre-output integration and verification. This phased temporal pattern aligns with the cognitive logic of “perception-comprehension-expression” in translation processes.

In human-machine collaborative translation, translators’ cognitive load demonstrates distinct phase-specific patterns. Observational data reveals that during the initial task phase, participants primarily rely on machine-generated translations for preliminary output, with generally lower eye-tracking metrics indicating relatively low cognitive load. As translation progresses, particularly when handling challenging text segments, cognitive load shows significant escalation, manifested through increased review attempts and sustained fixation duration. This progression reflects translators’ need to allocate additional cognitive resources to compensate for machine translation limitations while utilizing automated assistance. Specifically, in later stages where machine-generated translations contain errors or incompleteness, translators must deploy greater cognitive resources for correction and optimization, resulting in intensified cognitive load. Correspondingly, elevated review attempts and sustained fixation duration reveal heightened mental effort, while increased saccade frequency indicates frequent attentional switching between different text sections.

According to Table 2, the eye tracking data (fixation time, number of regressions, number of fixations, number of saccades) differed between task type, simple task, and text type (human translation and human-computer collaborative translation). In the simple task, the human translation value of fixation time was 31 (30∼33), and the human-computer collaborative translation value was 21 (20∼23), *Z* = −6.724, *P* < 0.01. It can be seen that the fixation time in the human translation task is significantly longer than that in the human-robot collaborative translation task. This may indicate that human translators need more time to understand and generate the translated content during the translation process, resulting in longer fixation times. The human translation value of the number of regressions was 6 (5∼7), and the human-computer collaborative translation value was 3 (3∼4), *Z* = −5.945, *P* < 0.01. The number of regressions in human translation is significantly higher than that in human-computer collaborative translation. This may be due to the fact that human translators frequently review the original text during the translation process to confirm the accuracy of the translation or to understand some complex content. The human translation value of fixation points was 22 (21∼24), and the human-computer collaborative translation value was 19 (17∼20), *Z* = −5.961, *P* < 0.01. The number of fixations in human translation is significantly higher than in human-machine collaborative translation. This may mean that during human translation, the translator’s visual focus is more widely distributed, requiring frequent attention to different areas of text. The human translation value of saccade number was 12 (11∼14), and the human-computer collaborative translation value was 8 (7∼9), *Z* = −6.449, *P* < 0.01. The number of saccades in human translation is also significantly higher than that of human-computer collaborative translation, indicating that human translators need to switch their attention more frequently during the translation process, and human-computer collaborative translation reduces this switching with the help of tools.

**TABLE 2:** Task type and text type differences for eye movement data (simple).

Task type	Gaze time (simple)	Return times (simple)	Number of focus points (simple)	Eye twitch frequency (simple)
Human translation	31 (30∼33)	6 (5∼7)	22 (21∼24)	12 (11∼14)
Human-machine collaboration	21 (20∼23)	3 (3∼4)	19 (17∼20)	8 (7∼9)
*Z*	−6.72	−5.95	−5.96	−6.45
*P*	<0.01	<0.01	<0.01	<0.01

According to Table 3, in the medium task, the human translation value of fixation time was 51 (50∼52), and the human-computer collaborative translation value was 41 (40∼42), *Z* = −6.794, *P* < 0.01. The fixation time of human translation is significantly longer than that of human-robot collaborative translation. For tasks of medium complexity, human translators may take more time to process and understand text of medium difficulty. The number of regressions was 8 (7∼8), and the value of human-computer collaborative translation was 6 (6∼7), *Z* = −5.463, *P* < 0.01, and the number of regressions of human translators was still higher than that of human-computer collaborative translation, which was related to the increase of task complexity and the need for translators to review and check in the process. The number of fixations was 34 (33∼35) for human translation and 28 (27∼29) for human-computer collaborative translation, *Z* = −6.807, *P* < 0.01, and the number of fixations for human translation was significantly higher than that for human-computer collaborative translation. This suggests that human translators may be more distracted during the translation process, focusing on different parts of the text. The human translation value of saccade was 18 (18∼19), and the human-computer collaborative translation value was 15 (14∼16) *Z* = −6.784, *P* < 0.01. The number of saccades in human translation is significantly higher than that in human-computer collaborative translation. Translators may need more saccades to adjust their attention when faced with a moderately difficult task.

**TABLE 3:** Differences between task types and text types for eye tracking data (medium).

Task type	Gaze time (medium)	Return times (medium)	Number of focus points (medium)	Eye twitch frequency (medium)
Human translation	51 (50∼52)	8 (7∼8)	34 (33∼35)	18 (18∼19)
Human-machine collaboration	41 (40∼42)	6 (6∼7)	28 (27∼29)	15 (14∼16)
*Z*	−6.79	−5.46	−6.81	−6.78
*P*	<0.01	<0.01	<0.01	<0.01

As indicated in Table 4, the fixation time values in complex tasks were 87 (85∼90) for human translation and 70 (68∼73) for human-computer collaborative translation, *Z* = −6.775, *P* < 0.01. As the complexity of the task increases, the gaze time of a human translator increases significantly, likely because complex texts require more time to understand, analyze, and translate. The human translation value of the number of regressions was 19 (18∼20), and the human-computer collaborative translation value was 15 (15∼16), *Z* = −6.584, *P* < 0.01. In complex tasks, the number of regressions by human translators is significantly higher than that of human-computer collaborative translation, which may be related to the need to repeatedly review and revise the translated content for complex tasks. The human translation value of fixation points was 57 (55∼60), and the human-computer collaborative translation value was 52 (50∼55), *Z* = −5.144, *P* < 0.01. The number of fixations in human translation is higher than that in human-machine collaborative translation, indicating that human translators are likely to process more areas of text when faced with complex tasks. The human translation value of saccade was 29 (28∼30), and the machine co-translation value was 23 (22∼24), *Z* = −6.806, *P* < 0.01. The number of saccades of human translators is significantly higher than that of human-computer collaborative translation, indicating that the attention of human translators switches between different parts more frequently in complex tasks.

**TABLE 4:** Task type and text type differences for eye movement data (complex).

Task type	Gaze time (complex)	Return times (complex)	Number of focus points (complex)	Eye twitch frequency (complex)
Human translation	87 (85∼90)	19 (18∼20)	57 (55∼60)	29 (28∼30)
Human-machine collaboration	70 (68∼73)	15 (15∼16)	52 (50∼55)	23 (22∼24)
*Z*	−6.78	−6.58	−5.14	−6.80
*P*	<0.01	<0.01	<0.01	<0.01

In summary, regardless of the difficulty of the task, whether it is easy, moderate, or complex, human translation is significantly higher than human-computer collaborative translation in all eye movement indicators (fixation time, number of regressions, number of fixations, and number of saccades). Fixation time increases with the complexity of the task, and the fixation time of human translators is especially long in complex tasks, which may indicate that human translators need more time to think when translating complex texts. The difference in the number of regression glances and saccades also increases with the increasing complexity of the task, and the phenomenon of frequent regression glances and saccades by human translators may reflect their repeated checks and adjustments to the text during the translation process. Overall, the tabular data suggests that human translators typically require more eye tracking resources than human-robot collaborative translation when performing translation tasks, especially when facing more complex tasks, and the cognitive load of human translators may be greater.

### 4.2 Distribution of cognitive load in human-computer collaborative translation

**FIGURE 5:**
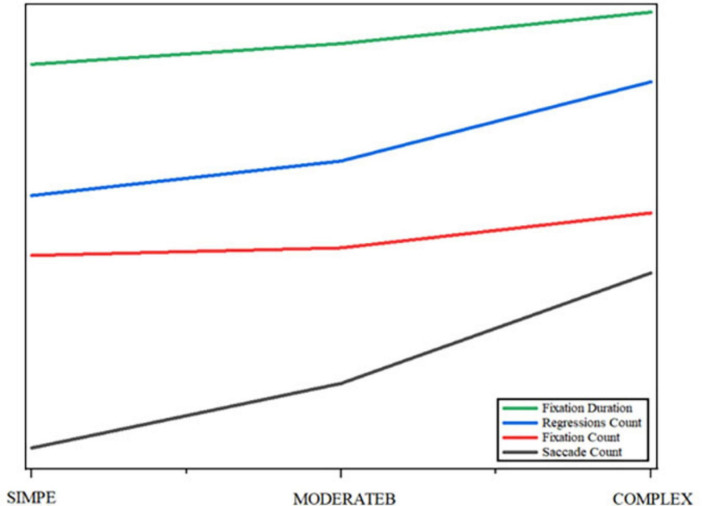
Trend fitting results for eye tracking data.

According to Figure 5, in the process of human-computer collaborative translation, the cognitive load shows obvious phased changes. At the beginning of the task, participants mainly rely on machine translation to generate preliminary translations, and the cognitive load at this stage is low. With the deepening of the translation task, especially when dealing with the more difficult parts, the cognitive load increases significantly, which is mainly reflected in the increase of the number of regressions and fixation time. This change shows that translators rely on machine translation while still consuming additional cognitive resources to correct inaccuracies in machine translation. Specifically, in the later stages of text processing (e.g., when dealing with complex sentences or proper nouns), machine translation results may be incorrect or incomplete, and at this time, translators need more cognitive resources to correct and optimize, resulting in an increase in the concentration of cognitive load. At this stage, the increase in the number of regressions and fixation time reflects the translator’s mental burden, and the number of saccades increases accordingly, indicating that the translator’s attention is shifted between multiple parts of the translation.

The study clearly reveals the dynamic interplay of three types of cognitive load in human-machine collaborative translation, with each type demonstrating distinct behavioral patterns. Intrinsic cognitive load is directly driven by task complexity, manifesting as significantly increased fixation durations and regression counts when processing complex texts, reflecting the inherent difficulty of handling linguistic structures and terminology. Extraneous cognitive load is tied to tool-use efficiency: in human translation, the lack of assistive tools leads to frequent saccades and a wider distribution of fixations, whereas in human-machine collaborative translation, initial reliance on machine output reduces extraneous load. However, poor-quality machine translations introduce additional corrective burdens. Germane cognitive load is concentrated in the revision phase of collaborative translation, where increased regressions and fixation durations reflect effective resource allocation as translators integrate machine output with human judgment.

## 5 Discussion

The intrinsic load still dominates in complex texts, but the preprocessing of machine translation can partially reduce the initial difficulty of understanding the source text. The extraneous load is reduced due to the intervention of tools - the machine-generated first draft avoids the repetitive work in human translation. However, low-quality machine output or interface switching may add new irrelevant burdens. The germane load is concentrated in the later revision stage by the translator, manifested as the evaluation and optimization of the machine translation. Although this process consumes resources, it is a necessary investment for improving translation quality.

In this study, we explored the differences in translators’ eye movement behaviors during human translation and human-computer collaborative translation, and verified the significant differences between the two translation modes through experimental data. The results show that the eye movement indicators (such as fixation time, number of regressions, number of fixations and saccades) of translators in the process of human translation are significantly higher than those of the human-computer collaborative translation mode, indicating that machine-assisted translation can effectively reduce the cognitive load of translators and improve translation efficiency. First, regarding the significant difference in fixation time, our results suggest that human translation tasks require longer concentrations. This is consistent with previous findings (Bacquelaine and Galvmango, 2024), which showed that translators in human translation often face more information processing pressure, especially when encountering complex grammatical structures and terminology, and the translator needs to review and refine the translated content repeatedly, and machine-assisted translation significantly reduces this burden by providing context and translation candidates on the fly (Wohltjen and Wheatley, 2024). Therefore, machine-assisted translation not only improves the efficiency of translation, but also improves the translator’s work experience. Second, the increase in the number of regressions and fixations further suggests that translators need more time to review and verify the accuracy of the translation during the human translation process, a phenomenon consistent with cognitive load theory (Mahanama et al., 2023). In human translation, the translator may be constantly looking back for the previous text due to the complexity of information processing to ensure the quality of the translation. In contrast, human-assisted translation reduces translators’ repeated reviews of previous content through automated feedback mechanisms, suggesting that machine-assisted translation can effectively reduce this cognitive fatigue (Tekwa, 2023).

## 6 Revelations and prospects

The findings of this study offer significant insights for translation instruction, professional practice, and the optimization of machine translation (MT) systems. (1) In terms of translation teaching, human-machine collaborative translation should be integrated into core courses, with a focus on post-editing skills. Students should be trained through real cases to identify typical machine error patterns, enhancing cognitive load management training-using strategies such as segmental processing and attention allocation, combined with eye-tracking feedback technology to help students visualize their cognitive resource consumption. (2) In the field of translation practice, a dynamic task allocation mechanism can be established, flexibly adjusting human-machine roles based on text complexity: high-complexity content should be led by humans, while basic parts can initially use machine translations. (3) In the design of the MT system, a “cognitive-friendly interface” needs to be developed: real-time annotation of translation risk points, automatic folding of repetitive sentence patterns to reduce visual interference, and allowing translators to customize information density. Implement “adaptive machine translation,” dynamically adjusting output based on eye movement data. (4) In terms of implementation: in the medium to short term (within 1 year), focus on developing teaching and training modules and interface optimization plugins; in the medium term (2–3 years), establish a two-way feedback mechanism between translators and machines, optimizing MT models through iterative practice data; in the long term (5 years or more), achieve an adaptive translation system based on cognitive science. (5) The machine translation system (especially when handling complex contexts or texts with dense professional terminology) may not provide entirely accurate suggestions, and translators still need to rely on their professional judgment. Efforts can be made to explore how to optimize the system to offer precise suggestions in more scenarios.

The limitations of this study lie in the small sample size and its focus on specific types of translation tasks. Future research could expand the sample size to examine the impact of different language pairs and verify the effects of human-computer collaboration on the cognitive load of translators. Additionally, it could explore how individual differences among translators (such as experience levels and language proficiency) influence eye movement behavior during translation, thereby providing more comprehensive theoretical support. It is worth noting that while metrics like fixation duration can reflect changes in cognitive load, they cannot distinguish specific cognitive processes. Cross-validation using retrospective interviews or electroencephalography (EEG) data is necessary.

In summary, this study demonstrates that effective machine assistance and human-machine collaborative translation can reduce the cognitive load on translators while enhancing translation efficiency and quality. This has valuable implications for future translation practice and research. Future studies will further optimize translation tools, explore a broader range of translation scenarios and conditions, and continuously improve translation quality and productivity.

## Data Availability

The raw data supporting the conclusions of this article will be made available by the authors, without undue reservation.

## References

[B1] Bacquelaine F. Galvmango E. Z. (2024). Translating Ulyssei@s: Work in progress between general and specialised translation in light of NMT. *Cad. Trad.* 44:e95214. 10.5007/2175-7968.2024.e95214

[B2] Baumgarten S. (2024). “Welcome to the translation machine! translation labour in times of techno-triumphalism,” in *Book: Translation and neoliberalism*, eds Daghigh A. J. Shuttleworth M. (Cham: Springer), 169–185.

[B3] Chang C. Y. Chen I. F. (2023). Translation directionality and the inhibitory control model: A machine learning approach to an eye-tracking study. *Front. Psychol.* 14:1196910. 10.3389/fpsyg.2023.1196910 37205087 PMC10187886

[B4] Chen S. Kruger J. L. (2024). Visual processing during computer-assisted consecutive interpreting: Evidence from eye movements. *Interpreting* 26 231–252. 10.1075/intp.00104.che 33486653

[B5] Cui Y. Liu X. Wang W. (2024). “Reliability of effort indicators for human translation and post-editing of machine translation: An eye tracking and keylogging study involving student translators,” in *Proceedings of the International Symposium on Emerging Technologies for Education*, (Springer: Singapore), 207–218.

[B6] Fredrick D. R. Craven L. (2025). Lexical diversity, syntactic complexity, and readability: A corpus-based analysis of ChatGPT and L2 student essays. *Front. Educ.* 10, 1616935–1616935. 10.3389/feduc.2025.1616935

[B7] Ge D. Y. Xiang W. Zhu S. Yao X. F. (2023). Hand-eye calibration method and machine vision research based on sensor network. *J. Comput. Methods Sci. Eng.* 23 1–14. 10.3233/JCM-226846

[B8] Kasper R. Motiejūnien J. Pataien I. Pataius M. Horbaauskien J. (2023). Is machine translation a dim technology for its users? An eye tracking study. *Front. Psychol.* 14:1076379. 10.3389/fpsyg.2023.1076379 36814649 PMC9939441

[B9] Liu Z. Riley P. Deutsch D. Lui A. Niu M. Shah A. (2024). “Beyond human-only: Evaluating human-machine collaboration for collecting high-quality translation data,” in *Proceedings of the Ninth Conference on Machine Translation*, (Miami, FL: Association for Computational Linguistics), 1095–1106.

[B10] Mahanama B. Sunkara M. Ashok V. Jayarathna S. (2023). “Disetrac: Distributed eye-tracking for online collaboration,” in *Proceedings of the 2023 Conference on Human Information Interaction and Retrieval*, (New York, NY: Machinery), 427–431.

[B11] Mahsuli M. M. Khadivi S. Homayounpour M. M. (2023). Lenm: Improving low-resource neural machine translation using target length modeling. *Neural Process. Lett.* 55 9435–9466. 10.1007/s11063-023-11208-1

[B12] Musk N. Meij S. V. D. (2024). Critical interactional strategies for selecting candidate translations in online translation tools in collaborative efl writing tasks. *Linguist. Educ.* 80:101290. 10.1016/j.linged.2024.101290

[B13] Odusami M. Maskeliūnas R. Damaeviius R. Misra S. (2024). Machine learning with multimodal neuroimaging data to classify stages of alzheimer’s disease: A systematic review and meta-analysis. *Cogn. Neurodyn.* 18 775–794. 10.1007/s11571-023-09993-5 38826669 PMC11143094

[B14] Pang J. Yang B. Wong D. F. Wan Y. Liu D. Chao L. S. (2024). Rethinking the exploitation of monolingual data for low-resource neural machine translation. *Comput. Linguist.* 50 25–47. 10.1162/coli_a_00496

[B15] Sweller J. (1988). Cognitive load during problem solving: effects on learning. *Cogn. Sci.* 12, 257–285. 10.1207/s15516709cog1202_4

[B16] Tekwa K. (2023). Process-oriented collaborative translation within the training environment: Comparing team and individual trainee performances using a video-ethnography approach. *Educ. Inf. Technol.* 29 6443–6469. 10.1007/s10639-023-12046-3

[B17] Wang R. Chen J. (2024). Nmthc: A hybrid error correction method based on a generative neural machine translation model with transfer learning. *BMC Genomics* 25:573. 10.1186/s12864-024-10446-4 38849740 PMC11157743

[B18] Wang Y. Daghigh A. J. (2023). Cognitive effort in human translation and machine translation post-editing processes. *Int. J. Interpretation Transl.* 21 139–162. 10.1075/forum.22009.wan 33486653

[B19] Wohltjen S. Wheatley T. (2024). Interpersonal eye-tracking reveals the dynamics of interacting minds. *Front. Hum. Neurosci.* 18:1356680. 10.3389/fnhum.2024.1356680 38532792 PMC10963423

